# Development of an Indirect ELISA and a Microsphere Detection Test Strip for the Detection of *Spirometra mansoni*

**DOI:** 10.1155/tbed/6256555

**Published:** 2026-07-27

**Authors:** Jie Hao, Hong Ying Zhang, Cheng Yue Cao, Gu Jin Guan, Zhong Quan Wang, Li Ma, Xi Zhang

**Affiliations:** ^1^ Department of Pathogen Biology, School of Basic Medical Sciences, Zhengzhou University, Zhengzhou 450001, Henan, China, zzu.edu.cn; ^2^ H&W Biotech Oy, Tekniikantie 12, Espoo 02150, Finland

**Keywords:** colour microsphere detection test strip, ELISA, excretory–secretory protein (ESP), pyruvate kinase (PK)

## Abstract

There is currently no highly sensitive and specific serological detection method for *Spirometra mansoni* infection, nor is there a rapid test strip available for onsite diagnosis. Therefore, in this study, an indirect enzyme‐linked immunosorbent essay (ELISA) method suitable for detecting *S. mansoni* was developed, and a colour microsphere test strip based on pyruvate kinase (PK) recombinant protein (r*Sm*PK) was developed. First, the excretory–secretory (ES) protein (ESP) of the plerocercoid was collected as the coating antigen to establish an ELISA method (*Sm*ES‐ELISA). Moreover, PK was screened as the target antigen, and an ELISA based on r*Sm*PK (r*Sm*PK‐ELISA) was performed. Finally, r*Sm*PK was labelled with NHS‐biotin, and NHS‐biotin‐r*Sm*PK was linked to coloured microspheres containing streptavidin (SA), successfully establishing a colour microsphere detection test strip. The optimal reaction conditions for *Sm*ES‐ELISA and r*Sm*PK‐ELISA were screened by systematically optimising conditions such as the coating concentration, serum dilution, blocking conditions and dilution of the enzyme‐labelled secondary antibody. Sensitivity testing revealed that *Sm*ES‐ELISA and r*Sm*PK‐ELISA still had good sensitivity at dilutions of 25,600 and 800 times, respectively. Specific detection was conducted using eight parasites that may be coinfected with *S. mansoni*, and the results showed that *Sm*ES‐ELISA did not exhibit cross‐reactivity, whereas r*Sm*PK‐ELISA only showed weak cross‐reactivity with *Clonorchis sinensis*. The colour microsphere detection test strip based on r*Sm*PK had good sensitivity but weak cross‐reactivity with *Hymenolepis nana*, *C. sinensis* and *Schistosoma japonicum*. However, in actual testing, there have been no reports of hosts simultaneously infected with these four parasites or even co‐infected with any two parasites. Therefore, the detection test strip constructed in this study still has high application value. *Sm*ES‐ELISA and r*Sm*PK‐ELISA compensate for the shortcomings of serological diagnostic methods for *S. mansoni* infection. Moreover, the colour microsphere detection test strip based on r*Sm*PK provides the possibility for large‐scale epidemiological investigations.

## 1. Introduction

The tapeworm *Spirometra mansoni* is an important zoonotic parasite. Adult *S. mansoni* worms live in the small intestines of definitive hosts, such as cats and dogs. The larvae (plerocercoids) can invade the human body and the bodies of all tetrapods and cause sparganosis, endangering human health and animal husbandry [1]. The life cycle of *S. mansoni* is a three‐host model: eggs shed from definitive hosts hatch into coracidia in water. After ingestion by primary intermediate copepods (such as Cyclops), coracidia mature into protocercariae. Infected copepods are then consumed by secondary intermediate hosts (tadpoles/frogs), where protocercariae develop into plerocercoids. If infected frogs are eaten by paratenic hosts (snakes and birds), larvae fail to mature into adults. They penetrate the intestinal wall and migrate to the abdominal cavity, muscles or subcutaneous tissues for survival [2]. Similarly, plerocercoid larva can also enter the human body and cause sparganosis, which manifests primarily as larval migration and can cause multiple lesions in locations such as subcutaneous tissues, oral and maxillofacial regions, eyes and the viscera and central nervous system, leading to tissue damage, blindness, paralysis and even death [3–5]. Sparganosis is distributed worldwide and is common in East and Southeast Asian countries. To date, more than 1300 cases of sparganosis have been reported in China, the greatest number of cases worldwide [6–8]. Sparganosis has not been given much attention by relevant departments for a long time due to the sporadic nature of cases of sparganosis, which is not often associated with outbreaks. Therefore, *S. mansoni* has become a neglected medical tapeworm.

The clinical diagnosis of sparganosis depends mainly on the biopsy of the plerocercoid. It is difficult to detect the plerocercoid, especially when the plerocercoid is parasitic in the viscera or central nervous system [9]. In practice, serological diagnosis is a good auxiliary diagnostic method, among which the indirect enzyme‐linked immunosorbent assay (ELISA) is highly sensitive and convenient and plays an important role in the diagnosis of parasitic diseases. Skotarek et al. [10] established a faecal antigen‐based ELISA (coproantigen ELISA) for the diagnosis of *Anoplocephala perfoliata* infection in horses, which showed high specificity. Figueroa‐Santiago et al. [11] established an ELISA method for the detection of *Fasciola hepatica* based on FhSAP2. Fhsap2‐ELISA is not only highly sensitive but also easy to purify and produce [11]. The indirect ELISA based on r*Tm*Adh3 established by Liu et al. [12] has good specificity for the early screening of polycephalum cerebri infection in sheep. However, there are few reports on the use of ELISAs for the detection of *S. mansoni*, and only some studies have reported problems such as high cross‐reactivity and low sensitivity [13]. Therefore, establishing a highly sensitive, specific and simple ELISA method for the diagnosis of sparganosis and the detection of *S. mansoni* infection in cats, dogs and other pets is particularly important. The excretory–secretory (ES) proteins (ESPs) of parasites are excreted by parasites during infection and are directly exposed to the host immune system; thus, these proteins can be used as ideal diagnostic antigens [14]. ELISAs based on ES proteins have been successfully constructed for the detection of a variety of parasites, such as *Haemonchus contortus*, *Fasciola hepatica* metacercariae and *Brugia malayi* [15]. However, an ELISA‐based diagnostic method for the detection of *S. mansoni* ES has not been established, and this gap needs to be filled urgently.

In addition, high‐quality candidate proteins are essential for the construction of specific ELISAs [11]. Previous studies have shown that pyruvate kinase (PK) is a key rate‐limiting enzyme in the glycolytic pathway and plays important roles in parasite growth, development and reproduction [16, 17]. Among them, PK 1 (*Sm*PK1) is a key gene affecting the growth and development of *S. mansoni* [18]. It can be used as an ideal target for ELISA detection. To date, there have been no reports of indirect ELISA methods based on PK. Therefore, the development of an indirect ELISA based on this protein can enrich the detection targets of *S. mansoni*.

Although the indirect ELISA detection method has high sensitivity and specificity, it requires specialised detection equipment and takes a long time; thus, point‐of‐care diagnosis cannot be achieved in the field [19]. In recent years, test strips using coloured microspheres, fluorescent microspheres, colloidal gold and other materials have been developed; these strips do not need to rely on special instruments and can be completed quickly, thus playing an important role in the field of parasitic disease diagnosis [20]. Fu et al. [21] developed an immunochromatographic test strip (ICS) using colloidal gold labelled with the ESP of *Trichinella spiralis* muscle larvae, which also has good sensitivity and specificity compared with ELISA. Moreover, it has achieved good results in field applications [21]. Wang et al. [22] successfully developed a colloidal gold ICS for the rapid and simple diagnosis of *Paragonimiasis skrjabini*, with high sensitivity, high specificity and good stability. Xu et al. [23] developed a GICA strip for the diagnosis of schistosomiasis, which has the advantages of high sensitivity, simple operation and low cost and is suitable for large‐scale screening of livestock in endemic areas of schistosomiasis. For sheep fascioliasis, a research team found that the CGIA detection method based on the rCatL1D protein has high sensitivity and specificity and can be used as a rapid diagnostic tool for this disease [24]. However, there is no rapid test strip for the field diagnosis of sparganosis and *S. mansoni* infection in pets. Therefore, in this study, a point‐of‐care test strip for the field diagnosis of *S. mansoni* infection based on the indirect ELISA method was developed to provide technical support for the diagnosis of sparganosis and the epidemiological surveillance of *S. mansoni* infection in pets such as cats and dogs.

Specifically, the objectives of this study include the following: (1) to establish an indirect ELISA based on the ESP and PK recombinant protein (r*Sm*PK) of *S. mansoni* and (2) to develop a colour microsphere detection test strip for the field detection of *S. mansoni* infections using r*Sm*PK.

## 2. Materials and Methods

### 2.1. Parasites and Experimental Animals

The plerocercoid was isolated from *Zaocys dhumnades*, Changsha, China. The sparganum was identified as *S. mansoni* based on the mitochondrial cytochrome c oxidase subunit 1 (*cox*1) gene according to a previously described method [6, 25]. In the laboratory, the plerocercoid larvae were preserved and passaged by feeding the head of the collected plerocercoid (about the first‐fifth of the worm) to Kunming mice [26]. Forty female BALB/c mice, aged 6–8 weeks, were purchased from the Laboratory Animal Centre of Henan Province for the collection of plerocercoid infection serum, protein immune serum and control serum. Female adult domestic cats were selected to collect sera from *S. mansoni*‐infected and control cats.

### 2.2. Serum Collection

For negative serum collection, female BALB/c mice without plerocercoid infection, aged 6–8 weeks, were fed for 1 week, and blood samples were collected from the tail vein. Serum was separated by incubation at 37°C for 2 h, centrifuged at 3000 r/min for 15 min, and stored at −20°C until use. For positive serum collection, female BALB/c mice, 6–8 weeks old, were infected with plerocercoid, blood samples were collected at 1‐week intervals after infection and serum was separated as described above and stored at −20°C until use. Six‐month‐old female domestic cats were infected with plerocercoid larvae at a dosage of 2 per animal, and successful infection was confirmed when the eggs were found after 10 days of continuous stool examination [26]. Approximately 45 days after infection, venous blood was collected to serve as *S. mansoni*‐infected serum, which was separated as described above and stored at −20°C until use. In addition, for cross‐reaction testing, we collected sera from other parasite‐infected patients with possible co‐infection with *S. mansoni*, including *Hymenolepis nana*, *Clonorchis sinensis*, *Schistosoma japonicum*, *Trichinella spiralis*, *Cryptosporidium parvum*, *Giardia duodenale*, *Toxoplasma gondii* and *Leishmania donovani*.

### 2.3. Collection of SmES

After being thoroughly washed with sterile saline and serum‐free RPMI‐1640 medium containing 100 U/mL penicillin and 100 U/mL streptomycin, the plerocercoid larvae were inoculated into 75 cm^2^ Petri dishes at a density of one parasite per mL and cultured for 24 h at 37°C in the presence of 5% CO_2_ [26]. At the end of the culture, the culture medium containing ES products was filtered through a 0.2 μm filter membrane into a 50 mL conical tube and centrifuged at 10,000 × *g* for 10 min at 4°C. The supernatant was ultrafiltered through a 10 kDa ultrafiltration tube and fully replaced with PBS. Finally, the ES protein was diluted to a concentration of 0.396 mg/mL and stored at −20°C until use. The collected plerocercoid ESPs were identified by SDS–PAGE and western blot analysis of the infected serum.

### 2.4. Cloning and Identification of rSmPK

The cloning, expression and identification of *Sm*PK1 were performed according to the methods described by Zhou et al. [18], and the specific steps are briefly described as follows. Plerocercoid cDNA was used as a template for PCR amplification and sequencing verification, after which the PCR product was ligated with the cloning vector pMD19‐T and transformed into DH5α competent cells. After further sequencing verification, the target gene was recovered by double digestion, ligated with the expression vector pQE‐80L and transformed into BL21 competent cells for the successful construction of the pQE‐80L/*Sm*PK/BL21 recombinant strain. The recombinant strain was then incubated at 25°C with 0.2 mM IPTG for 13 h to induce the expression of r*Sm*PK. The recombinant protein was purified using Ni NTA purification resin, and its purity was determined by SDS–PAGE according to the manufacturer’s instructions. The concentration of the recombinant protein was determined using a BCA protein assay kit. The prepared r*Sm*PK samples were separated by SDS–PAGE and then transferred to polyvinylidene fluoride (PVDF) membranes. After transfer, the samples were blocked with 5% skim milk powder at 37°C for 1 h and then cleaned with Tris‐buffered saline (TBST) containing Tween 20 for 5 min. The cells were incubated with a 1:100 dilution of anti‐r*Sm*PK polyclonal antibody, plerocercoid‐infected serum and BALB/c mouse blank serum as primary antibodies at 4°C overnight and with a 1:5000 dilution of goat anti‐mouse IgG‐HRP as a secondary antibody at 37°C for 1 h. Finally, 3,3′‐diaminobenzidine (DAB) chromogenic solution was used for colour development, and images were taken.

### 2.5. Preparation of rSmPK Polyclonal Antibodies

After emulsification of the purified r*Sm*PK with Freund’s complete adjuvant at a 1:1 ratio, the mice were immunised at a dose of 20 μg per mouse for the first time and then immunised with Freund’s incomplete adjuvant every 2 weeks for a total of four times. Finally, the purified recombinant protein was used to immunise BALB/c mice to prepare polyclonal antibodies.

### 2.6. Establishment of Indirect ELISA Methods Based on SmES and rSmPK

#### 2.6.1. Determination of the Coating Concentration and Optimal Serum Reaction Concentration

Using the chequerboard method, *Sm*ES and r*Sm*PK were serially diluted in ELISA coating solution at eight different concentrations (0.25, 0.5, 1.0, 1.5, 2.0, 2.5, 5.0 and 10.0 μg/mL). At the same time, the test serum was diluted at ratios of 1:100, 1:200, 1:400, 1:800 and 1:1600 and added to the microplate at a concentration of 100 μL/well. The other operation steps were carried out according to routine ELISA procedures [12]. The *P/N* value was calculated, and the optimal antigen coating concentration and the serum reaction concentration were selected as those that resulted in an OD_450 nm_ > 1 and the greatest *P/N* value.

#### 2.6.2. Determination of Optimal Sealing Conditions

*Sm*ES and r*Sm*PK were coated according to optimal coating conditions. The blocking times of *Sm*ES and r*Sm*PK were optimised at gradients of 30, 60, 90 and 120 min at 37°C. The remaining procedures were performed according to routine ELISA procedures [12]. The optimal sealing condition was determined by calculating the *P*/*N* value.

#### 2.6.3. Determination of the Optimal Reaction Time

*Sm*ES and *rSm*PK were coated and blocked according to the best amount of the coating protein and the best blocking solution conditions. At 37°C, the *Sm*ES and r*Sm*PK were optimised according to four time gradients of 30, 60, 90 and 120 min, and the *P*/*N* value was calculated to determine the best reaction time for serum.

#### 2.6.4. Determination of the Optimal Reaction Concentration of the Enzyme‐Labelled Secondary Antibodies

*Sm*ES and *rSm*PK were coated according to the established optimal conditions, and negative and positive sera were added as primary antibodies for the reaction, which was repeated six times. *Sm*ES was diluted at a gradient of 1:4000, 1:5000, 1:6000, 1:8000, 1:10,000 and 1:12,000. *rSm*PK was diluted in a gradient of 1:3000, 1:4000, 1:5000, 1:6000, 1:7000 and 1:8000. The optimal reaction concentration of the enzyme‐labelled secondary antibody was determined by calculating the *P*/*N* value.

#### 2.6.5. Determination of the Optimal Reaction Times of the Enzyme‐Labelled Secondary Antibodies

*Sm*ES and *rSm*PK were coated according to the determined optimal conditions, and after the addition of the primary antibody, the diluted enzyme‐labelled secondary antibody was added to each well and incubated according to the following conditions: 37°C for 30 min, 37°C for 60 min, 37°C for 90 min and 37°C for 120 min. ELISA was subsequently performed to determine the optimal reaction time of the enzyme‐labelled secondary antibody by calculating its *P*/*N* value.

#### 2.6.6. Measurement of Substrate Reaction Time

*Sm*ES and *rSm*PK were coated according to the determined optimal conditions, and each serum sample was detected by ELISA according to the optimal reaction conditions and optimal reaction time. Finally, 3,3′, 5,5′‐tetramethylbenzidine (TMB) was added to the substrate solution, which was divided into three groups according to the following conditions in the dark: 37°C for 5 min, 37°C for 10 min and 37°C for 15 min. The optimal substrate reaction time was determined by calculating the *P*/*N* value.

#### 2.6.7. Determination of Indirect ELISA Cut‐Off Values

According to the optimal reaction conditions and reaction time of each ELISA, 30 serum samples from normal mice were selected for the test. By measuring the OD_450 nm_ and calculating the mean ($\overset{―}{x}$) and standard deviation (SD) of each serum sample, the cut‐off value (cut‐off value = $\overset{―}{x}$ + 3 s) of the ELISA method was calculated. The determination standard of the ELISA detection method was determined according to the cut‐off value.

### 2.7. Detection of Sensitivity

The sparganum‐infected serum and BALB/c mouse blank serum samples were diluted to 1:100, 1:200, 1:400, 1:800, 1:1600, 1:3200, 1:6400, 1:12800 and 1:100, 1:200, 1:400, 1:800, 1:1600, 1:3200 and 1:6400, 1:12,800 and 1:25,600. The sensitivities of the established indirect ELISA method for *Sm*ES and r*Sm*PK were tested according to the optimal reaction conditions and reaction time of the established indirect ELISA method.

### 2.8. Detection of Specificity

According to the above experiments, the optimal reaction conditions and reaction time for each ELISA were determined. Blank serum from BALB/c mice was used as a control. Sera from plerocercoid, *S. mansoni*, *H. nana*, *C. sinensis*, *S. japonicum*, *T. spiralis*, *C. parvum*, *G. duodenale*, *T. gondii* and *L. donovani*. infections were used as the primary antibodies for ELISA detection. The negative and positive results were determined by measuring the OD_450 nm_ value and comparing it with the established cut‐off value to determine the specificity of the indirect ELISA.

### 2.9. Intra‐ and Interbatch Repeatability Tests

Intrabatch repeatability test: five serum samples from plerocercoid infections and three blank serum samples of BALB/c mice were collected. The same batch of coated and sealed microplate reaction plates was used, and indirect ELISAs based on the optimised *Sm*ES and r*Sm*PK proteins were performed. The mean and SD of the readings between repeated wells of each serum sample were calculated, and the coefficient of variation (CV) = (SD/mean) × 100% was calculated according to the statistical principle to test the intrabatch repeatability.

Interbatch repeatability test: five plerocercoid‐infected serum samples and three blank serum samples from BALB/c mice were collected. Based on the optimised indirect ELISA method for the *Sm*ES and r*Sm*PK proteins, two duplicate wells were established for each serum sample, and three independent repeated tests were performed by different researchers at different times. The mean of two replicate wells for each assay was calculated, the mean and SD of three independent assays of the same serum sample were sequentially calculated and the CV was calculated to determine the interbatch repeatability.

### 2.10. Indirect ELISA Detection for Batch Samples

Optimised *Sm*ES and *rSm*PK indirect ELISA methods were used to analyse 30 serum samples of plerocercoid infection and 30 blank serum samples of BALB/c mice under the same test conditions, and the sensitivity, specificity and accuracy were calculated.

### 2.11. Preparation of the Colour Microsphere Detection Test Strip

To achieve rapid detection of *S. mansoni* infection in the field, we developed a *S. mansoni* infection colour microsphere detection test strip. The strip was first labelled with NHS‐Biotin on *rSm*PK, and then, NHS‐Biotin‐*rSm*PK was connected to coloured microspheres modified with streptavidin (SA). Thus, *rSm*PK was successfully connected to the coloured microspheres, the IgY antibody was coupled to the activated coloured microspheres, and coloured microspheres coupled with the IgY antibody and coloured microspheres connected with *rSm*PK were sprayed on a microsphere pad. Finally, goat anti‐chicken IgY antibody was used as the quality control line and goat anti‐mouse IgG antibody was used as the detection line. Thus, a colour microsphere detection test strip for the detection of *S. mansoni* infection was established.

### 2.12. Sensitivity Testing of the Colour Microsphere Detection Test Strip

To evaluate the sensitivity of the established test strip, serum samples from plerocercoid‐infected mice and blank serum from BALB/c mice were serially diluted at ratios of 1:10, 1:20, 1:40, 1:80, 1:100, 1:200, 1:300 and 1:400. Colour development on the test line (T) was observed for each dilution.

### 2.13. Specificity Testing of the Colour Microsphere Detection Test Strip

Blank serum from BALB/c mice was used as a control to analyse serum samples infected with plerocercoid, *H. nana*, *C. sinensis*, *S. japonicum*, *T. spiralis*, *C. parvum*, *G. duodenale*, *T. gondii* and *L. donovani*. The colour development of the test line (T) was observed to test the cross‐reaction of the test strip.

### 2.14. Statistical Analysis

All analysis results were generated using GraphPad Prism 9.5, and differences were tested using one‐way analysis of variance (ANOVA), with significance marked as  ^∗^ *p* < 0.05,  ^∗∗^ *p* < 0.01,  ^∗∗∗^ *p* < 0.001,  ^∗∗∗∗^ *p* < 0.0001 and ns indicating no significant difference.

## 3. Results

### 3.1. Collection of SmES and Induced Expression of rSmPK

**Figure 1:**
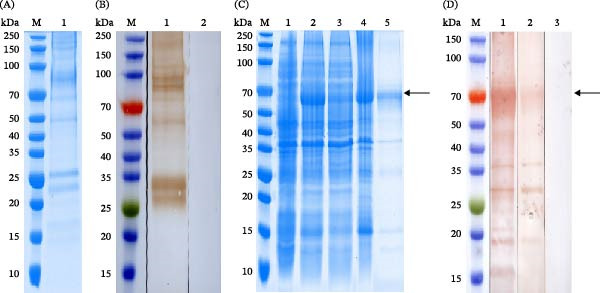
Collection and identification of *Sm*ES and induced expression and identification of *rSm*PK in *S. mansoni*. (A) SDS–PAGE analysis of *Sm*ES. M, marker; 1, *Sm*ES. (B) Western blot analysis of *Sm*ES. M, marker; 1, serum from *Sm*ES + infected mice; 2, serum from *Sm*ES + blank mice. (C) SDS–PAGE analysis of *rSm*PK. M, marker; 1, uninduced whole bacteria; 2, induced whole bacteria; 3, supernatant after ultrasonication; 4, precipitation after ultrasonication; 5, purified *rSm*PK. (D) Western blot analysis of *rSm*PK. M, marker; 1, *rSm*PK + anti‐*rSm*PK serum; 2, *rSm*PK + infected mouse serum; 3, *rSm*PK + blank mouse serum.

After being cultured in vitro, *Sm*ES was separated on a 10% polyacrylamide gel, and clear bands at 20, 25, 50, 70, 80 and 150 kDa were observed (Figure 1A). Western blot results revealed that the infected serum could better recognise *Sm*ES, whereas the blank serum did not react with *Sm*ES. These findings indicate that *Sm*ES has good specificity (Figure 1B). The recombinant plasmid PQE80L‐PK was induced by IPTG and purified by the Ni NTA purification resin, and a major band of ~65.24 kDa was detected by SDS–PAGE, which was consistent with the expected size (Figure 1C). Western blot results revealed that both anti‐*rSm*PK‐immune serum and anti‐rSmPK‐infected serum recognised *rSm*PK; however, blank serum did not react with *rSm*PK (Figure 1D).

### 3.2. Establishment of the Indirect ELISA Method

The square test was used to compare the OD_450 nm_ and *P*/*N* values of positive and negative serum samples (*P*: OD_450 nm_ value of positive serum; *N*: negative serum OD_450 nm_ value), and the *P*/*N* value was the greatest when the *Sm*ES coating concentration was 0.25 μg/mL and the serum dilution was 1:1600; thus, the optimal coating concentration of ES was determined to be 0.25 μg/mL (Figure 2A) and the optimal dilution of serum was 1:1600 (Figure 2B). When *Sm*ES was blocked at an optimal concentration of 1% BSA at 37°C for 30 min, the *P*/*N* value reached the maximum; therefore, the optimal blocking condition was chosen to be 1% BSA at 37°C for 30 min (Figure 2C). For the optimal reaction time of serum, according to the optimised conditions, *Sm*ES was coated at a concentration of 0.25 μg/mL, blocked with 1% BSA at 37°C for 30 min, fully washed with PBST, and positive or negative serum was added at 37°C for 30 min, after which the *P*/*N* value reached its maximum. Therefore, 30 min was selected as the optimal reaction time for serum (Figure 2D). In accordance with the above optimised conditions, *Sm*ES was coated, blocked and incubated with primary antibody, after which the enzyme‐labelled secondary antibody was gradually diluted. The results revealed that the *P*/*N* value of the *Sm*ES enzyme‐labelled secondary antibody reached a maximum when the dilution of the *Sm*ES enzyme‐labelled secondary antibody was 1:6000; thus, the optimal working concentration of the enzyme‐labelled secondary antibody was 1:6000 (Figure 2E). In accordance with the above optimised conditions, *Sm*ES was coated, blocked, incubated with primary antibody and diluted with a secondary antibody. After that, multiple groups of samples were incubated with a secondary antibody at different times. The results revealed that the *P*/*N* value of the enzyme‐labelled secondary antibody against *Sm*ES was the greatest when the sample was incubated at 37°C for 30 min; thus, 30 min was the best reaction time for the enzyme‐labelled secondary antibody (Figure 2F). To determine the substrate reaction time of *Sm*ES, after the sample was incubated with the enzyme‐labelled secondary antibody, the substrate solution was added. The *P*/*N* value of *Sm*ES reached a maximum when the colour developed at 37°C for 15 min in the dark; thus, 15 min in the dark was determined to be the best substrate reaction time (Figure 2G). Finally, the best indirect ELISA detection system for *Sm*ES was defined as follows: coating concentration: 0.25 μg/mL, serum dilution: 1:1600, blocking condition: 1% BSA blocking at 37°C for 30 min, serum reaction time: incubation at 37°C for 30 min, dilution of enzyme‐labelled secondary antibody: 1:6000, reaction time of enzyme‐labelled secondary antibody: samples were incubated at 37°C for 30 min, and the substrate reaction time was as follows: colour development at 37°C in the dark for 15 min.

Figure 2Establishment of an indirect ELISA for the detection of *S. mansoni*. (A) Determination of the optimal coating concentration of *Sm*ES. (B) Determination of the optimal concentration of serum for *Sm*ES. (C) Determination of the optimal blocking conditions of *Sm*ES. (D) Determination of the optimal reaction time of serum for *Sm*ES. (E) Determination of the optimal reaction concentration of the enzyme‐labelled secondary antibody for *Sm*ES. (F) Determination of the optimal reaction time of the enzyme‐labelled secondary antibody for *Sm*ES. (G) Determination of the reaction time of the *Sm*ES substrate. (H) Determination of the optimal concentration of *rSm*PK. (I) Determination of the optimal concentration of serum for *rSm*PK. (J) Determination of the optimal blocking conditions of *rSm*PK. (K) Determination of the optimal reaction time of serum for *rSm*PK. (L) Determination of the optimal reaction concentration of the enzyme‐labelled secondary antibody for *rSm*PK. (M) Determination of the optimal reaction time of the enzyme‐labelled secondary antibody for *rSm*PK. (N) Determination of the reaction time for the *rSm*PK substrate. Data are shown as mean ± standard deviation (SD). Error bars indicate standard deviation. *n* = 3 technical replicates per group.

**Figure:**
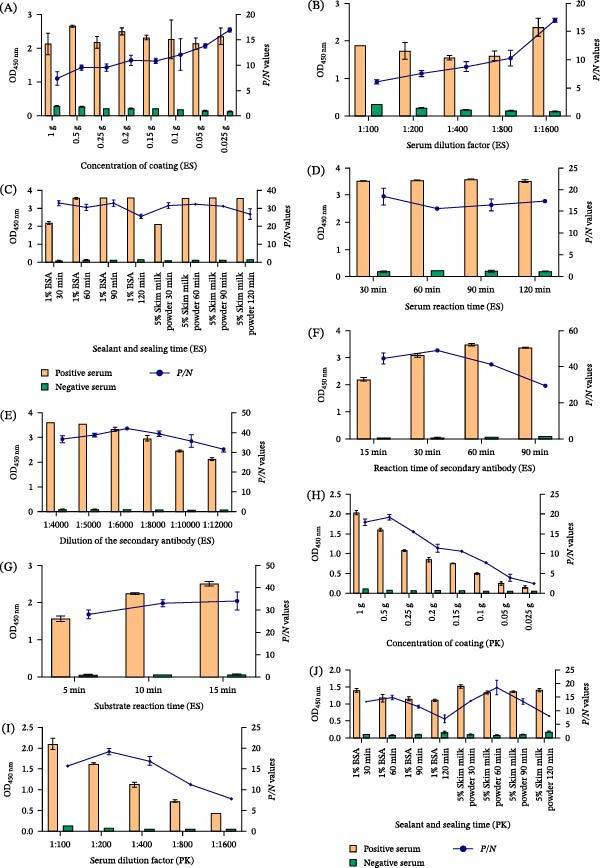

**Figure:**
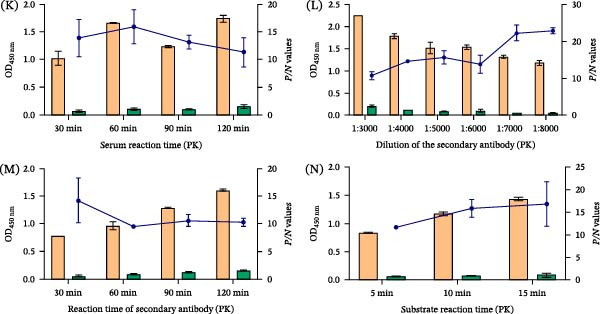

The optimal *rSm*PK indirect ELISA detection system was obtained according to the above methods. The optimal coating concentration was 5 μg/mL (Figure 2H), the optimal serum dilution was 1:200 (Figure 2I), the optimal blocking condition was 5% skim milk powder at 37°C for 60 min (Figure 2J), the optimal reaction time was 37°C for 60 min (Figure 2K), the optimal dilution of the HRP‐conjugated secondary antibody was 1:8000 (Figure 2L), the optimal reaction time of the HRP‐conjugated secondary antibody was 37°C for 30 min (Figure 2M) and the optimal reaction time of the substrate was 37°C for 15 min (Figure 2N).

### 3.3. Cut‐Off Value, Sensitivity and Specificity of Indirect ELISA

**Figure 3:**
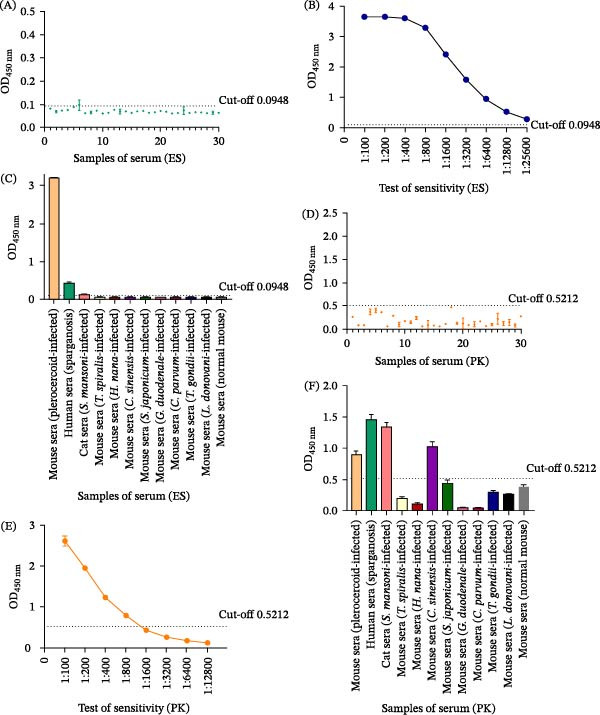
Cut‐off value, sensitivity and specificity experiments of the indirect ELISA method for detecting *S. mansoni*. (A) Determination of the cut‐off value of the *Sm*ES indirect ELISA detection method. (B) Sensitivity of the *Sm*ES indirect ELISA detection method. (C) Specificity of the *Sm*ES indirect ELISA detection method. (D) Determination of the cut‐off value of the r*Sm*PK indirect ELISA detection method. (E) Sensitivity of the r*Sm*PK indirect ELISA detection method. (F) Specificity of the r*Sm*PK indirect ELISA detection method. Data are shown as mean ± standard deviation (SD). Error bars indicate standard deviation. *n* = 3 technical replicates per group.

The established indirect ELISA detection method for *Sm*ES was used to detect 30 serum samples from normal mice, and the cut‐off value for ELISA detection was 0.0948, which was calculated according to the formula ‘$\overset{―}{x}$ + 3 s’, that is, when the OD_450 nm_ of the sample was ≥0.0948, the sample was judged to be positive (Figure 3A). Multiple dilution tests revealed that when the positive serum of plerocercoid was diluted to 1:25,600, the OD_450 nm_ value detected by the indirect ELISA method based on *Sm*ES was still greater than the critical value (0.0948), indicating that the method had good sensitivity (Figure 3B). To further verify the specificity of the indirect ELISA for *Sm*ES detection, we used the serum of plerocercoid and *S. mansoni* infections. In addition, serum from individuals infected with other common zoonotic parasites, such as *H. nana*, *C. sinensis*, *S. japonicum*, *T. spiralis*, *C. parvum*, *G. duodenale*, *T. gondii* and *L. donovani*, was tested for cross‐reaction, and the results revealed that the OD_450 nm_ values of these tested samples were less than 0.0948. These findings indicate that the indirect ELISA detection method based on *Sm*ES has good specificity (Figure 3C). Based on the same method, the OD_450 nm_ cut‐off value of the indirect ELISA established using *rSm*PK was 0.5212 (Figure 3D). Sensitivity testing revealed that when positive serum was diluted to 1:800, the detected OD_450 nm_ remained above the cut‐off value, indicating good sensitivity (Figure 3E). The results of the specificity test revealed that except for the cross‐reaction with *C. sinensis*, the OD_450 nm_ values of the other samples detected by the indirect ELISA method based on *rSm*PK were less than 0.5212, indicating good specificity (Figure 3F).

### 3.4. Repeatability Testing of Indirect ELISA

The optimised indirect ELISA detection method was used to test five positive serum samples infected with plerocercoid and three blank serum samples from the BALB/c mice under the same conditions. The results revealed that the CV of the intra‐ and interbatch repeatability test based on *Sm*ES for the indirect ELISA method ranged from 0.350% to 13.071% and from 3.778% to 13.564%, both of which were less than 15%, indicating good intra‐ and interbatch repeatability (Table 1). However, that of the intra‐ and interbatch repeatability test based on *rSm*PK ranged from 0.689% to 5.772% and from 4.018% to 14.703%, both of which were less than 15%, indicating good intra‐ and interbatch repeatability (Table 2). These results indicate that the indirect ELISA methods constructed based on *Sm*ES and *rSm*PK both have good repeatability.

**Table 1:** Intra‐ and interbatch reproducibility of indirect ELISA based on *Sm*ES.

Sample serial number	Intra‐assay		Interassay	
	Mean ± S	Coefficient of variation, Cv (%)	Mean ± *S*	Coefficient of variation, Cv (%)
P1	3.5 ± 0.105	3.026	3.403 ± 0.128	3.778
P2	3.077 ± 0.055	1.801	2.651 ± 0.359	13.564
P3	1.376 ± 0.019	1.42	1.428 ± 0.084	5.911
P4	3.238 ± 0.011	0.35	2.742 ± 0.295	10.772
P5	2.402 ± 0.06	2.503	2.206 ± 0.13	5.902
N1	0.04 ± 0.003	8.695	0.086 ± 0.007	8.795
N2	0.044 ± 0.003	8.121	0.084 ± 0.008	9.548
N3	0.043 ± 0.005	13.071	0.078 ± 0.006	8.569

**Table 2:** Intra‐ and interbatch reproducibility of indirect ELISA based on *Sm*PK.

Sample serial number	Intra‐assay		Interassay	
	Mean ± *S*	Coefficient of variation, Cv (%)	Mean ± *S*	Coefficient of variation, Cv (%)
P1	1.101 ± 0.021	1.906	0.96 ± 0.054	5.62
P2	0.78 ± 0.036	4.627	0.831 ± 0.122	14.703
P3	1.338 ± 0.042	3.151	1.355 ± 0.182	13.468
P4	1.453 ± 0.043	3.017	1.428 ± 0.086	6.033
P5	1.221 ± 0.008	0.689	1.09 ± 0.043	4.018
N1	0.139 ± 0.003	2.421	0.134 ± 0.007	5.403
N2	0.255 ± 0.014	5.772	0.261 ± 0.033	12.734
N3	0.217 ± 0.008	4.036	0.218 ± 0.031	14.543

### 3.5. Batch Sample Detection Using Indirect ELISA

**Figure 4:**
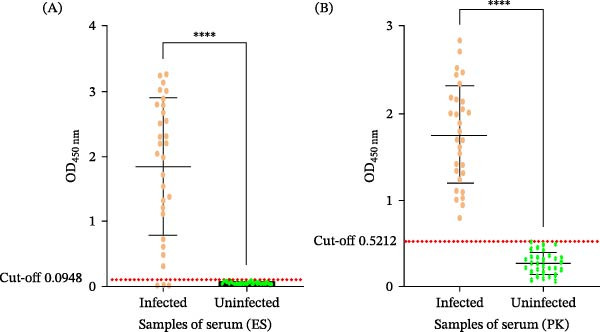
Batch sample detection of the indirect ELISA method for *S. mansoni*. (A) Batch sample detection using SmES‐ELISA. (B) Batch sample detection using rSmPK‐ELISA. Data are presented as mean ± SEM, each dot represents an individual biological replicate serum sample. *n* = 30 for both infected and uninfected groups.  ^∗∗∗∗^ *p* < 0.0001, significant difference between infected and uninfected groups.

Using the optimised ELISA detection method, 30 serum samples infected with plerocercoid and 30 blank serum samples under the same conditions were tested. The results revealed that the positive rate of infected serum detected by the indirect ELISA method based on *Sm*ES was 90% (27/30), whereas the positive rate of blank serum was 0% (0/30). According to the formula: sensitivity = TP/(TP + FN) × 100%, specificity = TN/(TN + FP) × 100%, and accuracy = (TP + TN)/(TP + TN + FP + FN) × 100% (true positive: TP, true negative: TN, false positive: FP, and false negative: FN), the sensitivity was calculated to be 90%, the specificity was 100% and the accuracy was 95%. Moreover, the difference in test results between infected and blank sera was significant (*p* < 0.05) (Table S1, Figure 4). The indirect ELISA method based on *rSm*PK defined the positive rate of infected serum to be 100% (30/30) and that of blank serum to be 0% (0/30), indicating that the sensitivity, specificity and accuracy were all 100%, and the difference in test results between infected serum and blank serum was significant (*p* < 0.05) (Table S1, Figure 4).

### 3.6. Construction of the Colour Microsphere Detection Test Strip for *S. mansoni*

**Figure 5:**
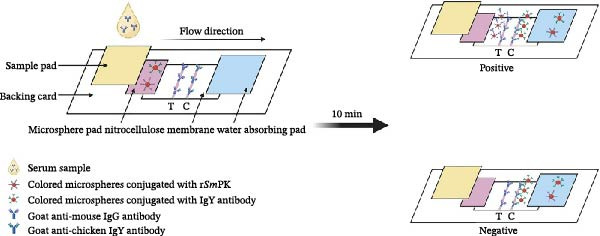
Schematic diagram of the construction of the *S. mansoni* colour microsphere detection test strip.

Given its good sensitivity and specificity and its ease of batch preparation, *rSm*PK was selected for the construction of a colour microsphere detection test strip for the detection of *S. mansoni* infection. First, coloured microspheres conjugated with IgY antibody and coloured microspheres labelled with *rSm*PK were sprayed on a microsphere pad. Goat anti‐chicken IgY antibody was used as a quality control line to coat the nitrocellulose (NC) membrane, and goat anti‐mouse IgG antibody was used as a detection line to coat the NC membrane. Then, the sample pad, microsphere pad, NC membrane and water absorber pad were assembled into test strips in sequence, and the test strips were put into the top cover and the bottom cover of the test paper to assemble a complete colour microsphere detection test strip for *S. mansoni* infection (Figure 5).

### 3.7. Detection of the Sensitivity of the Colour Microsphere Detection Test Strip

**Figure 6:**
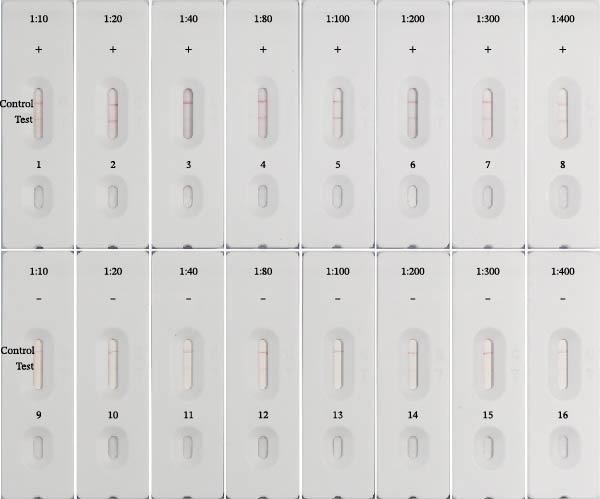
Detection of the sensitivity of the colour microsphere detection test strip for *S. mansoni* infection. Plerocercoid‐infected serum samples (1–8: 1:10, 1:20, 1:40, 1:80, 1:100, 1:200, 1:300 and 1:400) were added. Blank serum samples (9–16: 1:10, 1:20, 1:40, 1:80, 1:100, 1:200, 1:300 and 1:400) were added.

To test the sensitivity of the colour microsphere detection test strip, plerocercoid‐infected and blank sera were selected and diluted in a gradient of 1:10, 1:20, 1:40, 1:80, 1:100, 1:200, 1:300 and 1:400, and then the colour microsphere detection test strip was used to determine the results. When the infected serum was diluted to 1:400, the test line still showed a clear colour, whereas the blank serum did not show any colour, indicating that the test strip had good sensitivity (Figure 6).

### 3.8. Specificity of the Colour Microsphere Detection Test Strip

**Figure 7:**
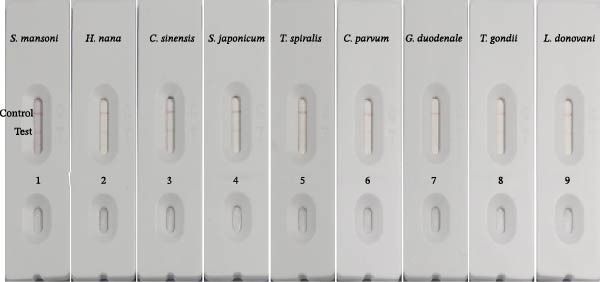
Cross‐reaction detection of the colour microsphere detection test strip for *S. mansoni* infection. 1–9: Serum samples infected with plerocercoid, *H. nana*, *C. sinensis*, *S. japonicum*, *T. spiralis*, *C. parvum*, *G. duodenale*, *T. gondii* and *L. donovani*.

To further validate the specificity of the coloured microsphere test strip, cross‐reaction tests were performed using serum samples infected with plerocercoid and other common zoonotic parasites, including *H. nana*, *C. sinensis*, *S. japonicum*, *T. spiralis*, *C. parvum*, *G. duodenale*, *T. gondii* and *L. donovani*. The results revealed that the strip test line (T) of the serum samples infected with plerocercoid showed an obvious colour, whereas the serum samples infected with other parasites showed no colour except for the serum samples infected with *H. nana*, *C. sinensis* and *S. japonicum* (Figure 7).

## 4. Discussion

Serological detection based on ELISA is highly important for the diagnosis of sparganosis, and cats and dogs are the common definitive hosts of *S. mansoni*, which plays an important role in the transmission of sparganosis [25, 27]. However, there is no sensitive and specific ELISA for the diagnosis of sparganosis and *S. mansoni* infection in pets such as cats and dogs. The need for rapid test strips for related onsite diagnosis represents another technical gap. Therefore, in this study, we first established an indirect ELISA method for the detection of *S. mansoni* antibodies using the ESP and the recombinant PK protein (r*Sm*PK) of *S. mansoni* and then developed a point‐of‐care test strip for the field diagnosis of *S. mansoni* infection based on the ELISA method to provide technical support for the diagnosis of sparganosis and the epidemiological surveillance of *S. mansoni* infection in pets such as cats and dogs.

Previous studies have shown that ESPs are secreted by parasites during infection. These proteins are directly expressed by the host’s immune system and are closely related to the host’s immune response, making them ideal candidates for serological diagnosis [14, 28, 29]. PK is a key rate‐limiting enzyme in the glycolytic metabolic pathway and plays a crucial role in the development, survival and reproduction of parasites [16, 17]. Among them, PK 1 of *S. mansoni* has been shown to be an important gene affecting the growth and development of *S. mansoni* [18]. Therefore, in this study, the ESP (*Sm*ES) and PK 1 (r*Sm*PK) of *S. mansoni* were selected for the construction of an indirect ELISA. In the construction of ELISA detection methods, a series of conditions, such as coating concentration, serum dilution, blocking conditions, serum reaction time, enzyme‐labelled secondary antibody dilution, enzyme‐labelled secondary antibody reaction time and substrate reaction time, can impact the detection effect of the ELISA [30–32]. In this study, different gradients were designed to optimise the system according to these influencing factors, and the ratio of the OD_450 nm_ between positive and negative sera (*P*/*N*) was used as the judgement standard [32]. The optimal conditions were ultimately determined as follows: for the *Sm*ES‐ELISA, optimal detection was achieved with *Sm*ES coating at a concentration of 0.25 μg/mL, serum dilution at 1:1600, blocking with 1% BSA at 37°C for 30 min, primary antibody incubation at 37°C for 30 min, enzyme‐conjugated secondary antibody dilution at 1:6000, secondary antibody incubation at 37°C for 30 min and substrate development in the dark at 37°C for 15 min; for the *rSm*PK‐ELISA, optimal detection was achieved with *rSm*PK coating at a concentration of 5 μg/mL, serum dilution at 1:200, blocking with 5% skim milk at 37°C for 60 min, primary antibody incubation at 37°C for 60 min, enzyme‐conjugated secondary antibody dilution at 1:8000, secondary antibody incubation at 37°C for 30 min and substrate development in the dark at 37°C for 15 min.

Then, the sensitivity, specificity, repeatability and batch samples of the *Sm*ES‐ELISA and r*Sm*PK‐ELISA were tested to determine the performance of the indirect ELISA. For sensitivity detection, the gradient dilution method is usually used [32]. Therefore, serum samples positive for plerocercoid were diluted to detect the lowest antibody concentration that could be stably detected by the *Sm*ES‐ELISA and r*Sm*PK‐ELISA to clarify the detection limit. The results revealed that the OD_450 nm_ values detected by the *Sm*ES‐ELISA and r*Sm*PK‐ELISA were still greater than the cut‐off values of 0.0948 and 0.5212 when the dilutions were 1:25,600 and 1:800, respectively. The sensitivity of the *Sm*ES‐ELISA was higher than those of *S. japonicum* and *Cysticercus pisiformis*, whereas the sensitivity of the r*Sm*PK‐ELISA was comparable to those of other parasites [32]. To test whether the *Sm*ES‐ELISA and r*Sm*PK‐ELISA cross‐reacted with other parasites, eight other parasites that may be coinfected with *S. mansoni* in the definitive host, namely, *H. nana*, *C. sinensis*, *S. japonicum*, *T. spiralis*, *C. parvum*, *G. duodenale*, *T. gondii* and *L. donovani*, were selected for specificity assays [33–35]. *Sm*ES‐ELISA did not show any cross‐reaction with other parasites, whereas r*Sm*PK‐ELISA only showed a weak cross‐reaction with *C. sinensis*. The reason for this result may be that r*Sm*PK still retained a small amount of complex proteins after induction and purification, which caused weak cross reactions [36]. In addition, to test the reliability and stability of the established ELISA method, to avoid bias caused by operation, reagent or instrument fluctuations, and to ensure repeatable and reliable test results, the repeatability tests for the *Sm*ES‐ELISA and r*Sm*PK‐ELISA were performed separately to evaluate the consistency of results of the same samples tested multiple times under the same conditions. The results revealed that the CV of the intra‐ and interbatch replicates of the *Sm*ES‐ELISA and r*Sm*PK‐ELISA were less than 15%, indicating good repeatability. To verify the high efficiency and applicability of the method, we further carried out batch sample detection experiments on the *Sm*ES‐ELISA and r*Sm*PK‐ELISA to confirm that they could meet the large‐scale requirements of actual detection. The results revealed that the sensitivities of *Sm*ES‐ELISA and r*Sm*PK‐ELISA were 90% and 100%, respectively; the specificities were 100% for both, and the was accuracies were 95% and 100%, respectively.

To achieve point‐of‐care detection for the diagnosis of *S. mansoni*, this study developed a colour microsphere detection test strip for *S. mansoni* based on r*Sm*PK. Colour microsphere detection test strips have the advantages of high sensitivity, clear and easy interpretation of results, simple and rapid operation and easy storage conditions [37]. Such strips have been widely used in the detection of bacterial, viral and other pathogenic microorganisms [37–39]. However, the application of colour microsphere detection test strip technology in parasite detection has not been previously reported. As mentioned earlier, r*Sm*PK was selected for the construction of a colour microsphere detection test strip for *S. mansoni* infection due to its good sensitivity and specificity, as well as its ease of batch preparation. The results of the sensitivity test revealed that the test line could still show obvious colour when the serum positive for plerocercoid was diluted to 1:400, indicating that the test strip had good sensitivity, which was comparable to those of test strips developed for the detection of *E. granulosus* and other parasites [23, 40, 41]. In the specificity test, none of the samples showed colour except for weak staining with sera from individuals infected with *H. nana*, *C. sinensis* and *S. japonicum*. This may be due to the high antigenic homology of r*Sm*PK with *H. nana*, *C. sinensis* and *S. japonicum*, especially the common epitopes in the conserved structural protein regions, which led to the failure of r*Sm*PK to accurately recognise antibodies in serum [36]. Zhou et al. [18] performed conserved motif analysis on 172 PK genes from cestodes and trematodes. The results showed that all proteins encoded by these genes contain typical conserved domains, which are also found in *S. mansoni*, *H. nana*, *C. sinensis* and *S. japonicum*. This structural feature well explains the cross‐reactivity of *Sm*PK in immunological detection [18]. In the actual detection, hosts infected with *S. mansoni*, *H. nana*, *C. sinensis* and *S. japonicum* or any two co‐infections have not been reported. Therefore, the *S. mansoni* r*Sm*PK colour microsphere detection test strip constructed in this study still has a high application value.

Although the indirect ELISA detection method and colour microsphere detection test strip established in this study achieved the specific detection and rapid screening of antibodies, some limitations still exist and need to be further optimised in future studies. For example, owing to the objective limitations of the experimental conditions, the cross reactions of some species that are more closely related to *S. mansoni*, such as *Diphyllobothrium latum*, *Taenia solium* and *Echinococcus*, could not be tested. Therefore, the risk of false‐positive results cannot be completely ruled out. In addition, the POCT rapid test strip established in this study lacks detection data regarding storage stability, as relevant experiments could not be performed due to the limited research schedule. Meanwhile, key operational parameters, including the fading duration of background colouration and the optimal time window for result interpretation, have not been defined. This detection system can serve as an auxiliary clinical diagnostic tool for humans, dogs and cats. Nevertheless, only mouse serum samples were utilised for validation in the present study, resulting in limited sample representativeness. The obtained test results cannot fully and objectively reflect the actual clinical diagnostic performance of this assay in target hosts, namely, humans, dogs and cats. Restricted by the availability of clinical specimens, serum samples from naturally infected humans, dogs and cats could not be collected for systematic validation in this work. In follow‐up investigations, sufficient clinical serum samples from humans, dogs and cats will be recruited to conduct large‐scale blind clinical trials with expanded sample cohorts, so as to comprehensively evaluate the clinical diagnostic performance of the developed assay. Moreover, the colour of the microsphere test strip still has the problem of process control in terms of microsphere dispersion uniformity and spray film consistency; as a result, the colour intensities of different batches of products can easily differ. In future studies, time‐resolved fluorescence immunoassay (TRFIA) technology can be introduced to label secondary antibodies with rare‐earth ions (such as Eu^3+^) to take advantage of their long fluorescence lifetime and low background interference to expand the detection linear range, achieve accurate quantification of antibody concentration and provide a basis for the classification of infection degree. In the future, we will increase the collection of serum samples from individuals with sparganosis and comprehensively evaluate the sensitivity, specificity, positive predictive value and negative predictive value of the assay through large sample data to provide sufficient evidence for clinical promotion and to promote detection from laboratory research to practical application.

## 5. Conclusions

In this study, the coating concentration, serum dilution, blocking condition, serum reaction time, dilution and reaction time of an enzyme‐labelled secondary antibody were systematically optimised. Two serological detection methods, *Sm*ES‐ELISA and r*Sm*PK‐ELISA, were successfully constructed. A sensitivity test revealed that both the *Sm*ES ELISA and r*Sm*PK‐ELISA had good sensitivity. The *Sm*ES ELISA showed high specificity, and the r*Sm*PK‐ELISA showed only a weak cross‐reaction with *C. sinensis*. Both intra‐ and interbatch repeated tests showed that the two methods had good stability. In addition, based on the r*Sm*PK, a colour microsphere detection test strip for the field diagnosis of *S. mansoni* was constructed for the first time, providing the possibility for large‐scale epidemiological investigations and facilitating the realisation of precise sparganosis control.

## Author Contributions

Xi Zhang designed the present study. Jie Hao, Hong Ying Zhang, Cheng Yue Cao, Gu Jin Guan and Li Ma performed the experiments. Jie Hao analysed the data with the assistance of Xi Zhang. Xi Zhang, Zhong Quan Wang and Jie Hao wrote the manuscript.

## Funding

This work was supported by the National Key R&D Program of China (Grant 2024YFE0199100) and Fundamental Research Project of Key Scientific Research in Henan Province (Grant 24ZX003).

## Disclosure

All authors read and approved the final manuscript.

## Conflicts of Interest

The authors declare no conflicts of interest.

## Supporting Information

Additional supporting information can be found online in the Supporting Information section.

## Supporting information


**Supporting Information** Table S1. Batch sample detection using indirect ELISA methods for SmES and rSmPK.

## Data Availability

All the data supporting the findings of this article are included in the main article and its additional files.

## References

[1] Tokiwa T. , Fushimi M. , and Chou S. , et al.Aberrant Sparganosis in Cat Caused by *Spirometra mansoni* (Cestoda: Diphyllobothriidae): A Case Report, BMC Veterinary Research. (2024) 20, no. 1, 10.1186/s12917-024-03995-z. PMC11031918 38643141

[2] Kuchta R. , Phillips A. J. , and Scholz T. , Diversity and Biology of *Spirometra tapeworms* (Cestoda: Diphyllobothriidea), Zoonotic Parasites of Wildlife: A Review, International Journal for Parasitology: Parasites and Wildlife. (2024) 24, 10.1016/j.ijppaw.2024.100947, 100947. 39040598 PMC11261046

[3] Kim J. , Ock Y. , and Yang K. , et al.First Clinical Cases of Spirometrosis in Two Cats in Korea, The Korean Journal of Parasitology. (2021) 59, no. 2, 153–157, 10.3347/kjp.2021.59.2.153. 33951771 PMC8106990

[4] Liu S. , Zhou K. , and Gao F. , et al.Lactate Dehydrogenase Gene Family in *Spirometra mansoni* (Cestoda: Diphyllobothriidea)—Phylogenetic Patterns and Molecular Characteristics, Animals. (2023) 13, no. 23, 10.3390/ani13233642, 3642. 38066993 PMC10705530

[5] Xu F. F. , Chen W. Q. , and Liu W. , et al.Genetic Structure of *Spirometra mansoni* (Cestoda: Diphyllobothriidae) Populations in China Revealed by a Target SSR-Seq Method, Parasites & Vectors. (2022) 15, no. 1, 10.1186/s13071-022-05568-1. PMC9789593 36564786

[6] Kuchta R. , Kołodziej-Sobocińska M. , and Brabec J. , et al.Sparganosis (*Spirometra*) in Europe in the Molecular Era, Clinical Infectious Diseases. (2021) 72, no. 5, 882–890, 10.1093/cid/ciaa1036. 32702118

[7] Liu W. , Gong T. , and Chen S. , et al.Epidemiology, Diagnosis, and Prevention of Sparganosis in Asia, Animals. (2022) 12, no. 12, 10.3390/ani12121578, 1578. 35739914 PMC9219546

[8] Su X. Y. , Gao F. , and Wang S. Y. , et al.Annexin Gene Family in *Spirometra mansoni* (Cestoda: Diphyllobothriidae) and Its Phylogenetic Pattern Among Platyhelminthes of Medical Interest, Parasite. (2024) 31, 10.1051/parasite/2024034, 32. 38912916 PMC11195529

[9] Li W. , Song R.XX, and etal S. S. , Development of PCR, qPCR and LAMP Methods for the Detection of *Spirometra mansoni* (Cestoda: Diphyllobothriidae) in the Faeces of Dogs and Cats, Food and Waterborne Parasitology. (2025) 41, 10.1016/j.fawpar.2025.e00291, e00291. 41079405 PMC12508883

[10] Skotarek S. L. , Colwell D. D. , and Goater C. P. , Evaluation of Diagnostic Techniques for *Anoplocephala perfoliata* in Horses From Alberta, Canada, Veterinary Parasitology. (2010) 172, no. 3-4, 249–255, 10.1016/j.vetpar.2010.05.005. 20605685

[11] Figueroa-Santiago O. , Delgado B. , and Espino A. M. , *Fasciola hepatica* Saposin-Like Protein-2–Based ELISA for the Serodiagnosis of Chronic Human Fascioliasis, Diagnostic Microbiology and Infectious Disease. (2011) 70, no. 3, 355–361, 10.1016/j.diagmicrobio.2011.03.016. 21683266 PMC3260655

[12] Yinju L. , Wenhui L. , and Yue L. , et al.Research on the Establishment of an Indirect ELISA Diagnostic Method for Sheep Cerebral Multiceps Echinococcosis Using TmAdh3 Recombinant Protein, Chinese Veterinary Science. (2023) 53, 1082–1088.

[13] Cui J. , Li N. , and Wang Z. Q. , et al.Serodiagnosis of Experimental Sparganum Infections of Mice and Human Sparganosis by ELISA Using ES Antigens of *Spirometra mansoni* Spargana, Parasitology Research. (2011) 108, no. 6, 1551–1556, 10.1007/s00436-010-2206-2. 21181193

[14] Kořínková K. , Kovařčík K. , and Pavlíčková Z. , et al.Serological Detection of *Trichinella spiralis* in Swine by ELISA (Enzyme-Linked Immunosorbent Assay) Using an Excretory–Secretory (E/S) Antigen, Parasitology Research. (2008) 102, no. 6, 1317–1320, 10.1007/s00436-008-0911-x. 18278585

[15] Sadarama P. V. , Chirayath D. , and Pillai U. N. , et al.Canine Brugia Malayi Microfilarial Excretory/Secretory Protein-Based Antibody Assay for the Diagnosis of Brugian Filariasis in Dogs, Journal of Parasitic Diseases. (2019) 43, no. 4, 549–553, 10.1007/s12639-019-01125-3. 31749523 PMC6841868

[16] Xu D. , Liang J. , Lin J. , and Yu C. , PKM2: A Potential Regulator of Rheumatoid Arthritis via Glycolytic and Non-Glycolytic Pathways, Frontiers in Immunology. (2019) 10, 10.3389/fimmu.2019.02919. PMC6930793 31921178

[17] Zhang Y. , Xiao W. , Luo L. , Pang J. , Rong W. , and He C. , Downregulation of OsPK1, a Cytosolic Pyruvate Kinase, by T-DNA Insertion Causes Dwarfism and Panicle Enclosure in Rice, Planta. (2012) 235, no. 1, 25–38, 10.1007/s00425-011-1471-3. 21805151

[18] Zhou K. , Cao C. Y. , and Ru S. S. , et al.Transcriptomic Analysis Reveals That Pyruvate Kinase Potentially Plays a Key Role in the Differentiation of *Spirometra mansoni* Proglottids by Regulating the Glycolysis Pathway, PLOS Neglected Tropical Diseases. (2025) 19, no. 10, 10.1371/journal.pntd.0013570, e0013570. 41066361 PMC12510601

[19] Praud A. C. , Corde Y. , Drapeau A. , Meyer L. , and Garin-Bastuji B. , Assessment of the Diagnostic Sensitivity and Specificity of an Indirect ELISA Kit for the Diagnosis of *Brucella ovis* Infection in Rams, BMC Veterinary Research. (2012) 8, 10.1186/1746-6148-8-68. PMC3391974 22640401

[20] Rodriguez C. A. , Busselman R. E. , and Shen H. , et al.Validation of a Multiplex Microsphere Immunoassay for Detection of Antibodies to *Trypanosoma cruzi* in Dogs, Journal of Veterinary Diagnostic Investigation. (2023) 35, no. 6, 704–709, 10.1177/10406387231198525. 37670473 PMC10621557

[21] Fu B. Q. , Li W. H. , and Gai W. Y. , et al.Detection of Anti-Trichinella Antibodies in Serum of Experimentally-Infected Swine by Immunochromatographic Strip, Veterinary Parasitology. (2013) 194, no. 2–4, 125–127, 10.1016/j.vetpar.2013.01.036. 23485436

[22] Wang Y. , Wang L. , and Zhang J. , et al.Preparation of Colloidal Gold Immunochromatographic Strip for Detection of *Paragonimiasis skrjabini* , PLoS ONE. (2014) 9, no. 3, 10.1371/journal.pone.0092034, e92034. 24643068 PMC3958401

[23] Xu R. , Feng J. , and Hong Y. , et al.A Novel Colloidal Gold Immunochromatography Assay Strip for the Diagnosis of Schistosomiasis Japonica in Domestic Animals, Infectious Diseases of Poverty. (2017) 6, no. 1, 10.1186/s40249-017-0297-z, 84. 28388965 PMC5384140

[24] Xifeng W. , Mengfan Q. , and Kai Z. , et al.Development and Evaluation of a Colloidal Gold Immunochromatographic Assay Based on Recombinant Protein CatL1D for Serodiagnosis of Sheep Fasciolosis, Journal of Helminthology. (2019) 94, 10.1017/S0022149X19000919, e98. 31679525

[25] Zhang X. , Hong X. , and Liu S. N. , et al.Large-Scale Survey of a Neglected Agent of Sparganosis *Spirometra erinaceieuropaei* (Cestoda: Diphyllobothriidae) in Wild Frogs in China, PLOS Neglected Tropical Diseases. (2020) 14, no. 2, 10.1371/journal.pntd.0008019, e0008019. 32101542 PMC7043720

[26] Liu S. N. , Ru S. S. , and Wang R. J. , et al.Establishment of an Optimal In Vitro Culture System for Plerocercoid Larvae of the Medical Tapeworm *Spirometra mansoni* (Cestoda: Diphyllobothriidae), Acta Tropica. (2025) 271, 10.1016/j.actatropica.2025.107864, 107864. 41067514

[27] Hong S. , Shin H. , and Ryoo S. , et al.Prevalence of Parasitic Infections in Stray Cats From Gimpo-si, Gyeonggi-do, Korea, Parasites, Hosts and Diseases. (2025) 63, no. 2, 182–187, 10.3347/PHD.24061. 40452280 PMC12127814

[28] da Silva M. B. , Urrego J. R. , and Oviedo A. Y. , et al.The Somatic Proteins of *Toxocara canis* Larvae and Excretory-Secretory Products Revealed by Proteomics, Veterinary Parasitology. (2018) 259, 25–34, 10.1016/j.vetpar.2018.06.015. 30056980

[29] Memon M. A. , Tunio S. , and Abro S. M. , et al.A Comprehensive Review on *Haemonchus contortus* Excretory and Secretory Proteins (HcESPs): TH-9 Stimulated ESPs as a Potential Candidate for Vaccine Development and Diagnostic Antigen, Acta Tropica. (2024) 260, 10.1016/j.actatropica.2024.107462, 107462. 39527996

[30] Peng P. , Gao Y. , and Zhou Q. , et al.Development of an Indirect ELISA for Detecting Swine Acute Diarrhoea Syndrome Coronavirus IgG Antibodies Based on a Recombinant Spike Protein, Transboundary and Emerging Diseases. (2022) 69, no. 4, 2065–2075, 10.1111/tbed.14196. 34148289

[31] Qingli L. , Zhicheng W. , and Li M. , et al.Application of Recombinant Signal Protein 14-3-3 Indirect ELISA for the Diagnosis of *Schistosomiasis japonica* , Journal of Zoonotic Diseases in China. (2007) 23, no. 3, 231–235.

[32] Xifeng W. , Qingling M. , and Qiao J. , et al.Construction, Expression of MeCatL-B Fusion Gene of *Clonorchis sinensis* and Establishment of Its Indirect ELISA Detection Method, Chinese Journal of Preventive Veterinary Medicine. (2019) 41, 273–278.

[33] Magalhães F. C. , Resende S. D. , and Senra C. , et al.Accuracy of Real-Time Polymerase Chain Reaction to Detect *Schistosoma mansoni—*Infected Individuals From an Endemic Area With Low Parasite Loads, Parasitology. (2020) 147, no. 10, 1140–1148, 10.1017/S003118202000089X. 32484122 PMC10317740

[34] Swan C. , Phan T. , and McKew G. , Clinical Performance of Real-Time Polymerase Chain Reaction for *Strongyloides stercoralis* Compared With Serology in a Nonendemic Setting, The American Journal of Tropical Medicine and Hygiene. (2022) 107, no. 2, 355–358, 10.4269/ajtmh.21-1289. 35895584 PMC9393460

[35] Kamel H. H. , Elleboudy N. A. F. , and Hasan A. N. , et al.Nano Magnetic-Based ELISA and Nano Magnetic-Based Latex Agglutination Test for Diagnosis of Experimental Trichinellosis, Journal of Parasitic Diseases. (2023) 47, no. 2, 400–409, 10.1007/s12639-023-01583-w. 37193503 PMC10182192

[36] Jin Y. , Kim E.-M. , and Choi M.-H. , et al.Significance of Serology by Multi-Antigen ELISA for Tissue Helminthiases in Korea, Journal of Korean Medical Science. (2017) 32, no. 7, 10.3346/jkms.2017.32.7.1118, 1118. 28581268 PMC5461315

[37] Liang Z. , Peng T. , and Jiao X. , et al.Latex Microsphere-Based Bicolor Immunochromatography for Qualitative Detection of Neutralizing Antibody Against SARS-CoV-2, Biosensors. (2022) 12, no. 2, 10.3390/bios12020103, 103. 35200362 PMC8869495

[38] Jiang N. , Shi L. , and Lin J. , et al.Comparison of Two Different Combined Test Strips With Fluorescent Microspheres or Colored Microspheres as Tracers for Rotavirus and Adenovirus Detection, Virology Journal. (2018) 15, no. 1, 10.1186/s12985-018-0951-5. PMC5851252 29534739

[39] Shi F. , Tang Y. , and Xu Z. H. , et al.Visual Typing Detection of Brucellosis With a Lateral Flow Immunoassay Based on Coloured Latex Microspheres, Journal of Applied Microbiology. (2022) 132, no. 1, 199–208, 10.1111/jam.15240. 34319629

[40] Zhuo X. , Yu Y. , and Chen X. , et al.Development of a Colloidal Gold Immunochromatographic Strip Based on HSP70 for the Rapid Detection of *Echinococcus granulosus* in Sheep, Veterinary Parasitology. (2017) 240, 34–38, 10.1016/j.vetpar.2017.03.027. 28576342

[41] Meng X. , Yu Y. , and Ma D. , et al.Development of a Colloidal Gold Immunochromatographic Strip for the Rapid on-Site Detection of *Ecytonucleospora hepatopenaei* (EHP), Journal of Invertebrate Pathology. (2025) 209, 10.1016/j.jip.2024.108266, 108266. 39701445

