# Supplementary material for: Development of an Indirect ELISA and a Microsphere Detection Test Strip for the Detection of Spirometra mansoni
Source: Transbound Emerg Dis. 2026 Jul 27;2026:6256555. doi: 10.1155/tbed/6256555 (PMC13403222; doi:10.1155/tbed/6256555)
Supplement: Supplementary file 1 — Supporting Information Table S1. Batch sample detection using indirect ELISA methods for SmES and rSmPK. [file TBED-2026-6256555-s001.docx]

**Table S1.** Batch sample detection using indirect ELISA methods for *Sm*ES and r*Sm*PK.

| Samples of serum | Serum copies | *Sm*ES-ELISA | | r*Sm*PK-ELISA | |
| --- | --- | --- | --- | --- | --- |
|  |  | OD_450nm_ values (x±S) | Number positive/Total number | OD_450nm_ values (x±S) | Number positive/Total number |
| plerocercoid（+） | 30 | 1.84±1.05 | 27/30 | 1.76±0.56 | 30/30 |
| plerocercoid（-） | 30 | 0.038±0.022 | 0/30 | 0.26±0.13 | 0/30 |
